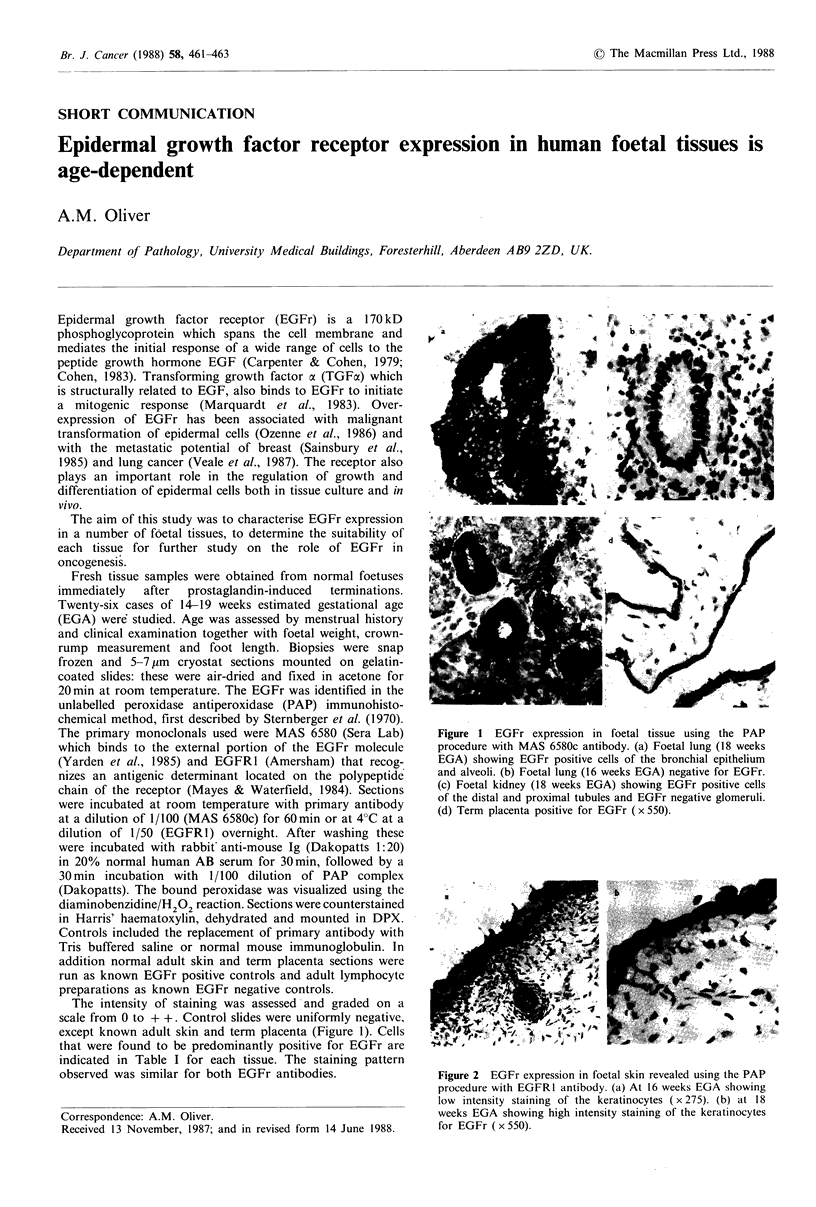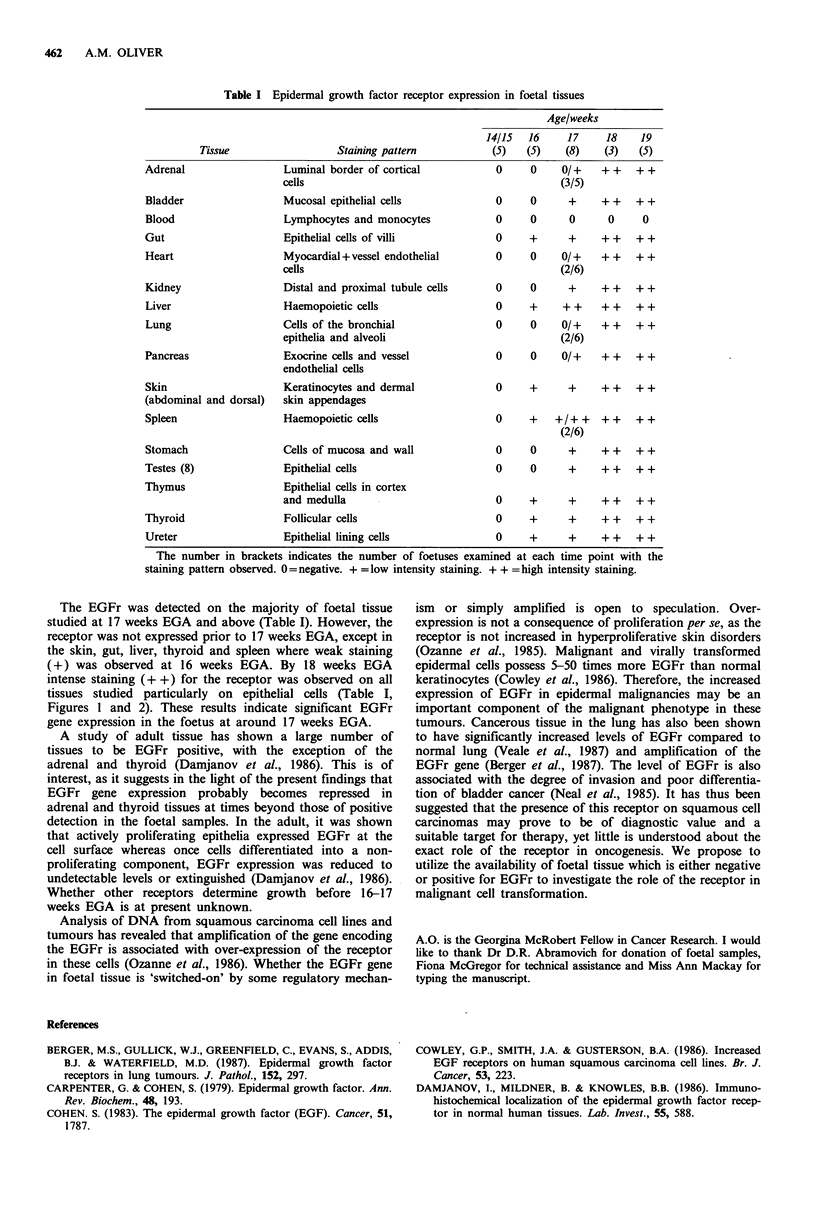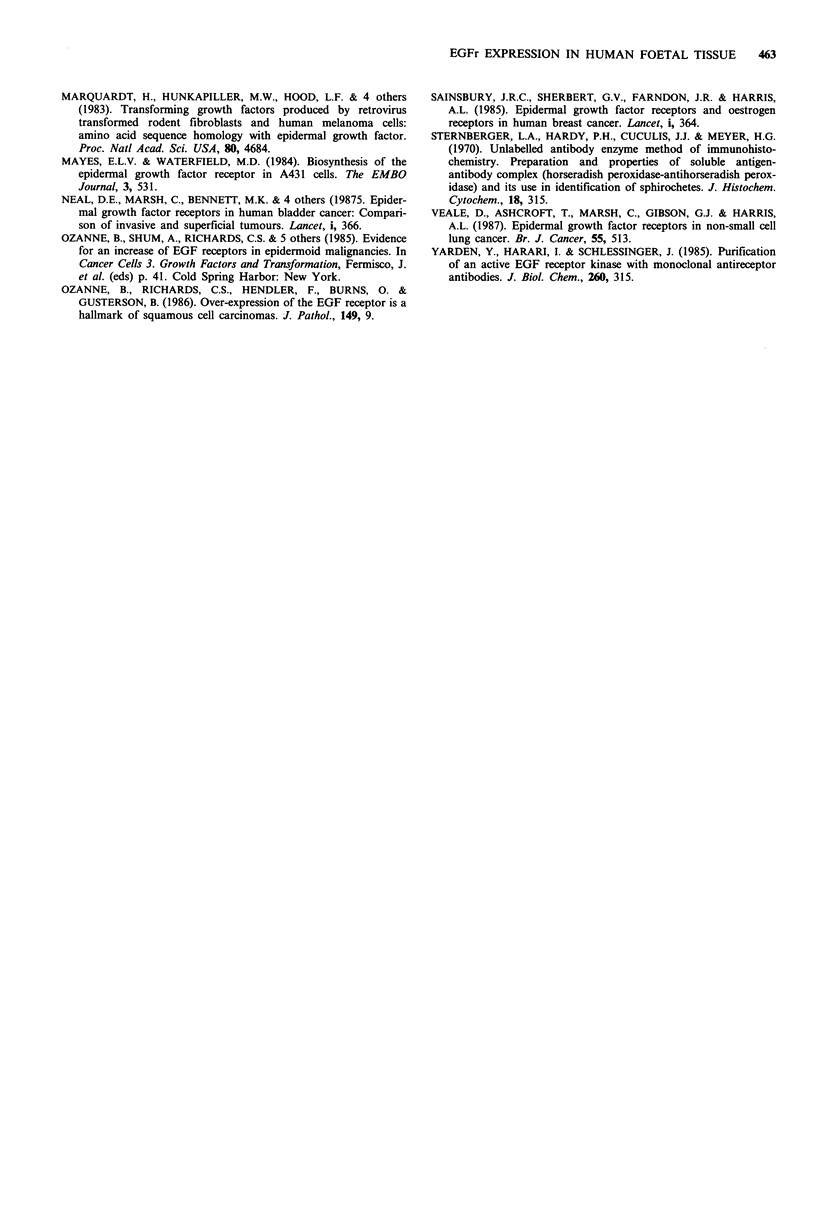# Epidermal growth factor receptor expression in human foetal tissues is age-dependent.

**DOI:** 10.1038/bjc.1988.241

**Published:** 1988-10

**Authors:** A. M. Oliver

**Affiliations:** Department of Pathology, University Medical Buildings, Aberdeen, UK.

## Abstract

**Images:**


*Br. J. Cancer* (1988) **58**, 461–463 © The Macmillan Press Ltd., 1988

SHORT COMMUNICATION

### Epidermal growth factor receptor expression in human foetal tissues is age-dependent

A.M. Oliver

*Department of Pathology, University Medical Buildings, Foresterhill, Aberdeen AB9 2ZD, UK.*

Epidermal growth factor receptor (EGFr) is a 170 kD phosphoglycoprotein which spans the cell membrane and mediates the initial response of a wide range of cells to the peptide growth hormone EGF (Carpenter & Cohen, 1979; Cohen, 1983). Transforming growth factor α (TGFα) which is structurally related to EGF, also binds to EGFr to initiate a mitogenic response (Marquardt *et al.*, 1983). Over-expression of EGFr has been associated with malignant transformation of epidermal cells (Ozenne *et al.*, 1986) and with the metastatic potential of breast (Sainsbury *et al.*, 1985) and lung cancer (Veale *et al.*, 1987). The receptor also plays an important role in the regulation of growth and differentiation of epidermal cells both in tissue culture and *in vivo*.

The aim of this study was to characterise EGFr expression in a number of foetal tissues, to determine the suitability of each tissue for further study on the role of EGFr in oncogenesis.

Fresh tissue samples were obtained from normal foetuses immediately after prostaglandin-induced terminations. Twenty-six cases of 14–19 weeks estimated gestational age (EGA) were studied. Age was assessed by menstrual history and clinical examination together with foetal weight, crown-rump measurement and foot length. Biopsies were snap frozen and 5–7 μm cryostat sections mounted on gelatin-coated slides: these were air-dried and fixed in acetone for 20 min at room temperature. The EGFr was identified in the unlabelled peroxidase antiperoxidase (PAP) immunohistochemical method, first described by Sternberger *et al.* (1970). The primary monoclonals used were MAS 6580 (Sera Lab) which binds to the external portion of the EGFr molecule (Yarden *et al.*, 1985) and EGFR1 (Amersham) that recognizes an antigenic determinant located on the polypeptide chain of the receptor (Mayes & Waterfield, 1984). Sections were incubated at room temperature with primary antibody at a dilution of 1/100 (MAS 6580c) for 60 min or at 4°C at a dilution of 1/50 (EGFR1) overnight. After washing these were incubated with rabbit anti-mouse Ig (Dakopatts 1:20) in 20% normal human AB serum for 30 min, followed by a 30 min incubation with 1/100 dilution of PAP complex (Dakopatts). The bound peroxidase was visualized using the diaminobenzidine/$H_2O_2$ reaction. Sections were counterstained in Harris' haematoxylin, dehydrated and mounted in DPX. Controls included the replacement of primary antibody with Tris buffered saline or normal mouse immunoglobulin. In addition normal adult skin and term placenta sections were run as known EGFr positive controls and adult lymphocyte preparations as known EGFr negative controls.

The intensity of staining was assessed and graded on a scale from 0 to + +. Control slides were uniformly negative, except known adult skin and term placenta (Figure 1). Cells that were found to be predominantly positive for EGFr are indicated in Table I for each tissue. The staining pattern observed was similar for both EGFr antibodies.

Correspondence: A.M. Oliver.
Received 13 November, 1987; and in revised form 14 June 1988.

**Figure 1** EGFr expression in foetal tissue using the PAP procedure with MAS 6580c antibody. (a) Foetal lung (18 weeks EGA) showing EGFr positive cells of the bronchial epithelium and alveoli. (b) Foetal lung (16 weeks EGA) negative for EGFr. (c) Foetal kidney (18 weeks EGA) showing EGFr positive cells of the distal and proximal tubules and EGFr negative glomeruli. (d) Term placenta positive for EGFr (×550).

**Figure 2** EGFr expression in foetal skin revealed using the PAP procedure with EGFR1 antibody. (a) At 16 weeks EGA showing low intensity staining of the keratinocytes (×275). (b) at 18 weeks EGA showing high intensity staining of the keratinocytes for EGFr (×550).

**Table I** Epidermal growth factor receptor expression in foetal tissues

| | | *Age/weeks* | | | | |
|---|---|---|---|---|---|---|
| *Tissue* | *Staining pattern* | *14/15* (*5*) | *16* (*5*) | *17* (*8*) | *18* (*3*) | *19* (*5*) |
| Adrenal | Luminal border of cortical cells | 0 | 0 | 0/+ (3/5) | + + | + + |
| Bladder | Mucosal epithelial cells | 0 | 0 | + | + + | + + |
| Blood | Lymphocytes and monocytes | 0 | 0 | 0 | 0 | 0 |
| Gut | Epithelial cells of villi | 0 | + | + | + + | + + |
| Heart | Myocardial + vessel endothelial cells | 0 | 0 | 0/+ (2/6) | + + | + + |
| Kidney | Distal and proximal tubule cells | 0 | 0 | + | + + | + + |
| Liver | Haemopoietic cells | 0 | + | + + | + + | + + |
| Lung | Cells of the bronchial epithelia and alveoli | 0 | 0 | 0/+ (2/6) | + + | + + |
| Pancreas | Exocrine cells and vessel endothelial cells | 0 | 0 | 0/+ | + + | + + |
| Skin (abdominal and dorsal) | Keratinocytes and dermal skin appendages | 0 | + | + | + + | + + |
| Spleen | Haemopoietic cells | 0 | + | +/+ + (2/6) | + + | + + |
| Stomach | Cells of mucosa and wall | 0 | 0 | + | + + | + + |
| Testes (8) | Epithelial cells | 0 | 0 | + | + + | + + |
| Thymus | Epithelial cells in cortex and medulla | 0 | + | + | + + | + + |
| Thyroid | Follicular cells | 0 | + | + | + + | + + |
| Ureter | Epithelial lining cells | 0 | + | + | + + | + + |

The number in brackets indicates the number of foetuses examined at each time point with the staining pattern observed. 0 = negative. + = low intensity staining. + + = high intensity staining.

The EGFr was detected on the majority of foetal tissue studied at 17 weeks EGA and above (Table I). However, the receptor was not expressed prior to 17 weeks EGA, except in the skin, gut, liver, thyroid and spleen where weak staining (+) was observed at 16 weeks EGA. By 18 weeks EGA intense staining (+ +) for the receptor was observed on all tissues studied particularly on epithelial cells (Table I, Figures 1 and 2). These results indicate significant EGFr gene expression in the foetus at around 17 weeks EGA.

A study of adult tissue has shown a large number of tissues to be EGFr positive, with the exception of the adrenal and thyroid (Damjanov *et al.*, 1986). This is of interest, as it suggests in the light of the present findings that EGFr gene expression probably becomes repressed in adrenal and thyroid tissues at times beyond those of positive detection in the foetal samples. In the adult, it was shown that actively proliferating epithelia expressed EGFr at the cell surface whereas once cells differentiated into a non-proliferating component, EGFr expression was reduced to undetectable levels or extinguished (Damjanov *et al.*, 1986). Whether other receptors determine growth before 16–17 weeks EGA is at present unknown.

Analysis of DNA from squamous carcinoma cell lines and tumours has revealed that amplification of the gene encoding the EGFr is associated with over-expression of the receptor in these cells (Ozanne *et al.*, 1986). Whether the EGFr gene in foetal tissue is 'switched-on' by some regulatory mechanism or simply amplified is open to speculation. Over-expression is not a consequence of proliferation *per se*, as the receptor is not increased in hyperproliferative skin disorders (Ozanne *et al.*, 1985). Malignant and virally transformed epidermal cells possess 5–50 times more EGFr than normal keratinocytes (Cowley *et al.*, 1986). Therefore, the increased expression of EGFr in epidermal malignancies may be an important component of the malignant phenotype in these tumours. Cancerous tissue in the lung has also been shown to have significantly increased levels of EGFr compared to normal lung (Veale *et al.*, 1987) and amplification of the EGFr gene (Berger *et al.*, 1987). The level of EGFr is also associated with the degree of invasion and poor differentiation of bladder cancer (Neal *et al.*, 1985). It has thus been suggested that the presence of this receptor on squamous cell carcinomas may prove to be of diagnostic value and a suitable target for therapy, yet little is understood about the exact role of the receptor in oncogenesis. We propose to utilize the availability of foetal tissue which is either negative or positive for EGFr to investigate the role of the receptor in malignant cell transformation.

A.O. is the Georgina McRobert Fellow in Cancer Research. I would like to thank Dr D.R. Abramovich for donation of foetal samples, Fiona McGregor for technical assistance and Miss Ann Mackay for typing the manuscript.

### References

BERGER, M.S., GULLICK, W.J., GREENFIELD, C., EVANS, S., ADDIS, B.J. & WATERFIELD, M.D. (1987). Epidermal growth factor receptors in lung tumours. *J. Pathol.*, **152**, 297.

CARPENTER, G. & COHEN, S. (1979). Epidermal growth factor. *Ann. Rev. Biochem.*, **48**, 193.

COHEN. S. (1983). The epidermal growth factor (EGF). *Cancer*, **51**, 1787.

COWLEY, G.P., SMITH, J.A. & GUSTERSON, B.A. (1986). Increased EGF receptors on human squamous carcinoma cell lines. *Br. J. Cancer*, **53**, 223.

DAMJANOV, I., MILDNER, B. & KNOWLES, B.B. (1986). Immunohistochemical localization of the epidermal growth factor receptor in normal human tissues. *Lab. Invest.*, **55**, 588.

MARQUARDT, H., HUNKAPILLER, M.W., HOOD, L.F. & 4 others (1983). Transforming growth factors produced by retrovirus transformed rodent fibroblasts and human melanoma cells: amino acid sequence homology with epidermal growth factor. *Proc. Natl Acad. Sci. USA*, **80**, 4684.

MAYES, E.L.V. & WATERFIELD, M.D. (1984). Biosynthesis of the epidermal growth factor receptor in A431 cells. *The EMBO Journal*, **3**, 531.

NEAL, D.E., MARSH, C., BENNETT, M.K. & 4 others (19875. Epidermal growth factor receptors in human bladder cancer: Comparison of invasive and superficial tumours. *Lancet*, **i**, 366.

OZANNE, B., SHUM, A., RICHARDS, C.S. & 5 others (1985). Evidence for an increase of EGF receptors in epidermoid malignancies. In *Cancer Cells 3. Growth Factors and Transformation*, Fermisco, J. *et al.* (eds) p. 41. Cold Spring Harbor: New York.

OZANNE, B., RICHARDS, C.S., HENDLER, F., BURNS, O. & GUSTERSON, B. (1986). Over-expression of the EGF receptor is a hallmark of squamous cell carcinomas. *J. Pathol.*, **149**, 9.

SAINSBURY, J.R.C., SHERBERT, G.V., FARNDON, J.R. & HARRIS, A.L. (1985). Epidermal growth factor receptors and oestrogen receptors in human breast cancer. *Lancet*, **i**, 364.

STERNBERGER, L.A., HARDY, P.H., CUCULIS, J.J. & MEYER, H.G. (1970). Unlabelled antibody enzyme method of immunohistochemistry. Preparation and properties of soluble antigen-antibody complex (horseradish peroxidase-antihorseradish peroxidase) and its use in identification of sphirochetes. *J. Histochem. Cytochem.*, **18**, 315.

VEALE, D., ASHCROFT, T., MARSH, C., GIBSON, G.J. & HARRIS, A.L. (1987). Epidermal growth factor receptors in non-small cell lung cancer. *Br. J. Cancer*, **55**, 513.

YARDEN, Y., HARARI, I. & SCHLESSINGER, J. (1985). Purification of an active EGF receptor kinase with monoclonal antireceptor antibodies. *J. Biol. Chem.*, **260**, 315.